# Management of Internal Root Resorption With Mineral Trioxide Aggregate in Mandibular Molar: A Case Report

**DOI:** 10.7759/cureus.96174

**Published:** 2025-11-05

**Authors:** Neelam Mittal, Chanda Dhakad, Shelly Sharma, Deepti Singh

**Affiliations:** 1 Department of Conservative Dentistry and Endodontics, Faculty of Dental Sciences, Institute of Medical Sciences, Banaras Hindu University (IMS-BHU), Varanasi, IND

**Keywords:** internal root resorption, mandibular molar, mta, non-surgical endodontic treatment, repair

## Abstract

Internal root resorption (IRR) is a pathological condition characterized by the loss of dentin due to the action of clastic cells triggered by pulpal inflammation. Most cases of internal root resorption occur in anterior teeth, primarily because of their susceptibility to trauma. However, it can also manifest in posterior teeth, often as a result of carious involvement of the pulp. Timely diagnosis, elimination of the underlying cause, and appropriate treatment of the resorbed root are essential for achieving a successful treatment outcome. Various techniques and materials have been employed to fill internal resorptive defects. Among these, mineral trioxide aggregate (MTA) demonstrates commendable properties, including biocompatibility, effective sealing ability, mechanical strength, and the capacity to promote healing of periradicular tissue. This case report presents the successful management and favorable outcome of internal root resorption in a mandibular molar through non-surgical endodontic treatment and repair of the resorptive defect using MTA.

## Introduction

Resorption is a physiologic or pathological process characterized by the loss of dentin, cementum, or bone [1]. Root resorption may follow mechanical, chemical, or thermal injury and is broadly classified as internal or external. Internal resorption is an inflammatory process originating within the pulp space, resulting in dentin loss and, in some cases, cementum involvement. It is categorized into two forms: internal root canal inflammatory resorption and internal root canal replacement resorption. Although its etiology is not fully understood, it has been associated with chronic pulpal inflammation, trauma, previous dental procedures, excessive heat, and cracked teeth [2]. The reported prevalence of internal resorption ranges from 0.01% to 55%, depending on pulpal status [3].

Histopathologically, internal resorption is characterized by the presence of clastic cells, such as odontoclasts, which actively resorb dentin from within the pulp chamber or canal. This process is typically initiated and sustained by chronic pulpal inflammation, leading to progressive loss of dentin and, in some cases, involvement of the adjacent cementum. The resorptive defect is usually lined by granulation tissue, which may further promote the activity of resorptive cells, highlighting the importance of early diagnosis and intervention to prevent extensive structural compromise.

Establishing a diagnosis for these lesions is often challenging, as conventional radiographs are usually insufficient. Early internal root radiolucencies are typically undetectable on standard two-dimensional X-rays due to their small size and inherent imaging limitations. Cone beam computed tomography (CBCT) offers a more advanced diagnostic approach, enabling earlier and more precise detection of such lesions [4,5].

A variety of materials have been proposed for the management of internal root resorption (IRR), including mineral trioxide aggregate (MTA), bioglass, super ethoxy benzoic acid (EBA), hydrophilic plastic polymer (2-hydroxyethyl methacrylate with barium salts), zinc oxide eugenol and zinc acetate cement, amalgam alloy, composite resin, and thermoplasticized gutta-percha applied through injection or condensation techniques. The prognosis of the affected tooth largely depends on the biomaterial selected for treatment. Among these, MTA remains the most widely used due to its superior biocompatibility, sealing properties, and ability to promote osteogenesis and cementogenesis. It is often followed by the thermoplasticized gutta-percha obturation technique [6].

## Case presentation

A 17-year-old male patient presented to the Department of Conservative Dentistry and Endodontics with a chief complaint of pain on biting, sensitivity to hot and cold temperatures, and food lodgment in the right lower back tooth region. The pain was sudden in onset and worsened while lying down at night. There was no significant past medical or relevant dental history. Clinical examination revealed deep occlusal caries in tooth 46, along with tenderness on percussion.

Periodontal probing depths around the affected tooth ranged from 2-3 mm, with grade 1 mobility. Pulp sensibility testing using cold (Endo-Ice, Maquira, Brazil) and electric pulp testing yielded no response, confirming loss of pulpal vitality. During access preparation, a perforation measuring approximately 1.5 × 1.8 mm was identified on the cervical third of the canal wall in the cervical third of the root.

**Figure 1:**
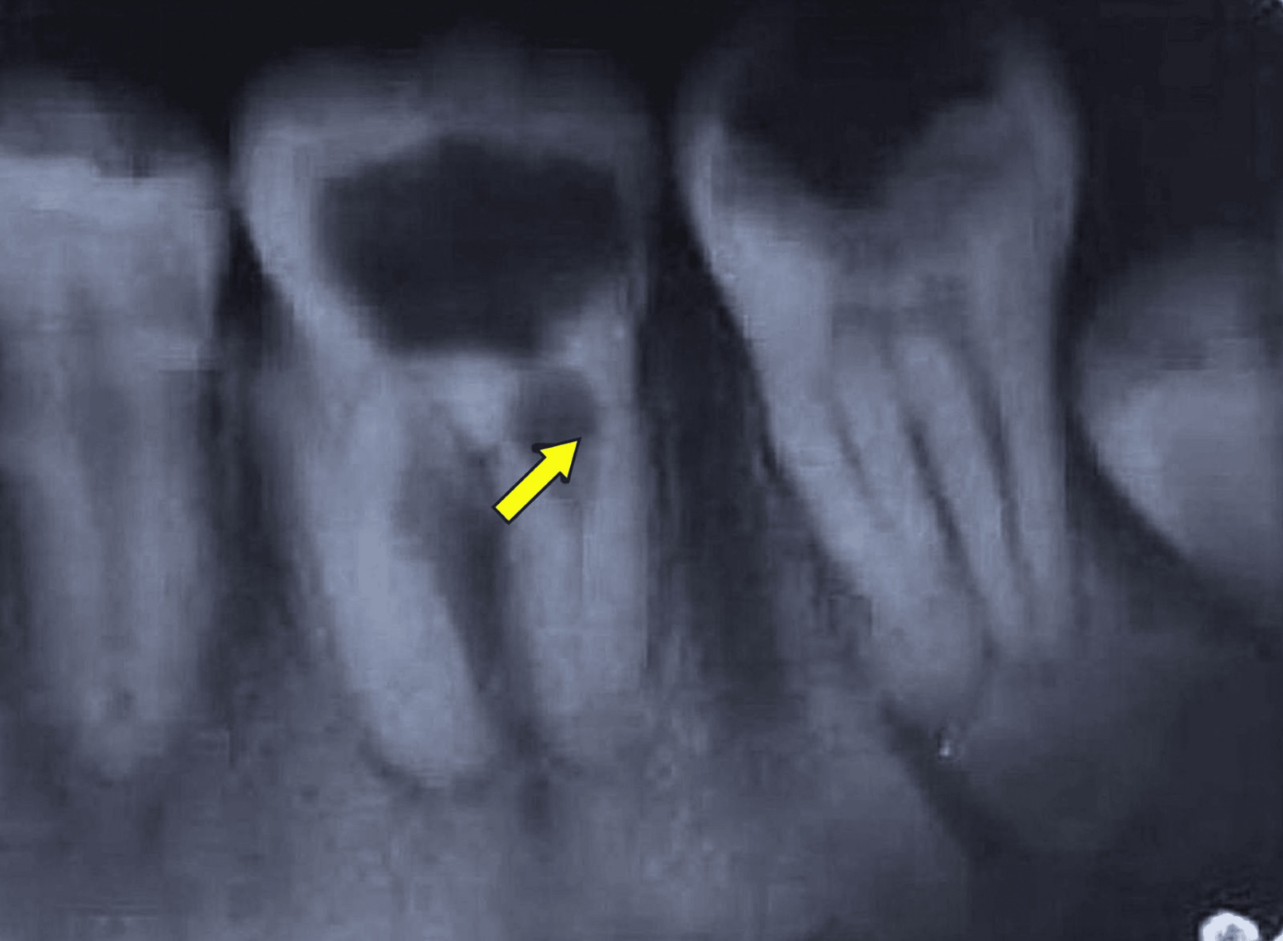
Preoperative periapical radiograph showing a well-defined internal resorptive defect (arrow) on the distal root of the mandibular first molar Note the loss of canal outline at the level of the cervical third, suggestive of active internal resorption.

**Figure 2:**
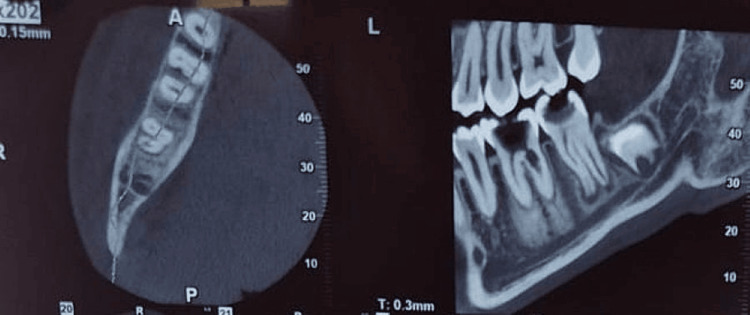
Preoperative CBCT revealed an oval-shaped radiolucency at the cervical third of the distal root and a furcal radiolucency CBCT: cone beam computed tomography

Radiographic examination of the preoperative radiograph (Figure 1) revealed a large coronal radiolucency consistent with deep occlusal caries, along with a small radiolucency in the cervical third of the distal root of tooth 46, suggestive of internal root resorption. Additionally, radiolucencies were evident in the periapical region with a periapical index (PAI) score of 4 and a furcation area, accompanied by bone loss. Preoperative CBCT examination revealed an oval-shaped radiolucency in the cervical third of the distal root, along with radiolucency in the furcal region (Figure 2).

Based on the clinical and radiographic findings, a diagnosis of irreversible pulpitis with apical periodontitis associated with internal resorption in relation to tooth 46 was established. The treatment plan, consisting of non-surgical endodontic therapy with resorption repair using mineral trioxide aggregate (MTA), was discussed with the patient, and treatment was initiated after obtaining informed consent.

First appointment

**Figure 3:**
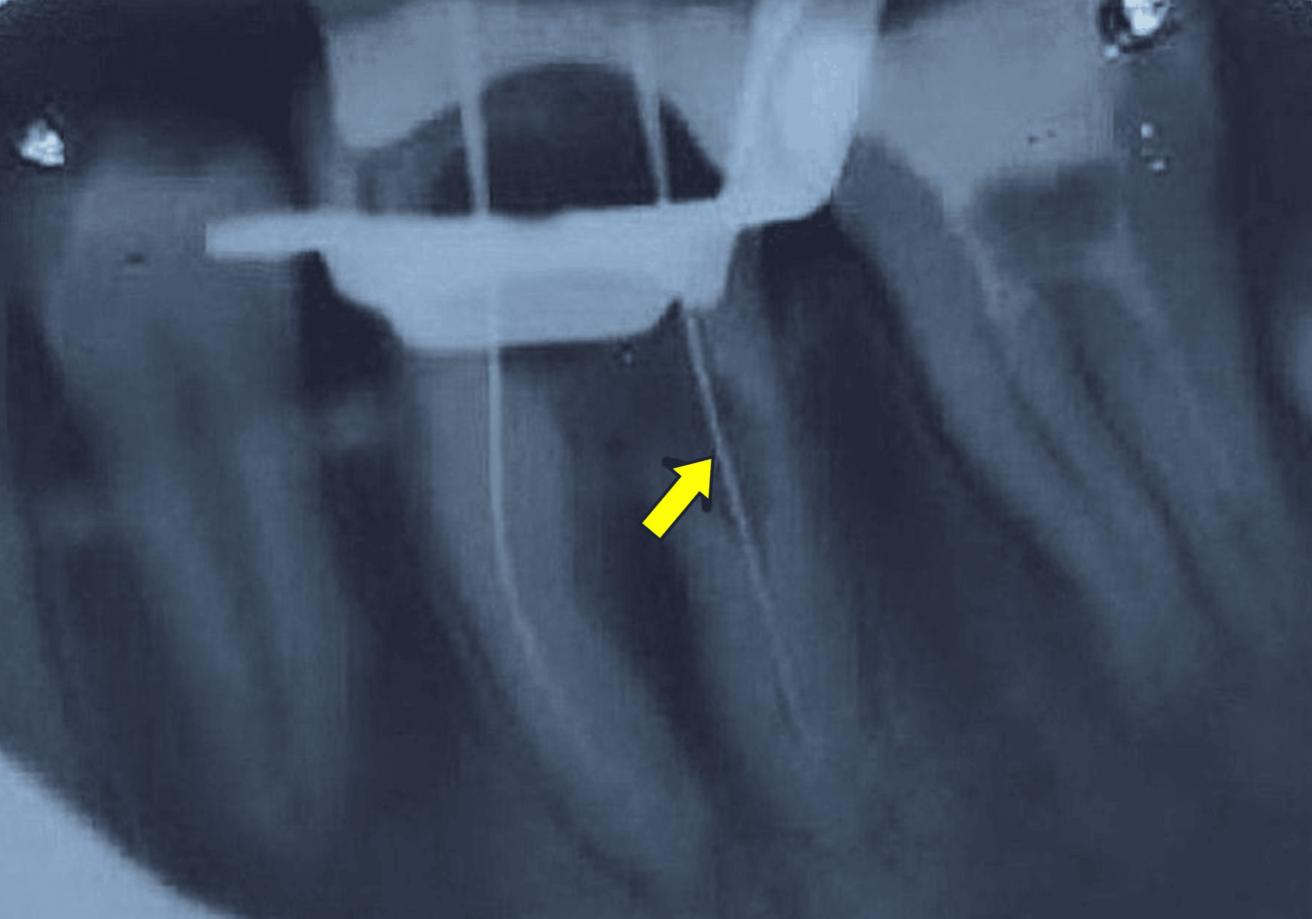
Working length radiograph (arrow indicates the resorptive area communicating with the canal wall)

Local anesthesia (2% lignocaine with 1:100,000 epinephrine) was administered, isolation was done with a rubber dam, and an access cavity was prepared using a high-speed round bur. All canals were identified and negotiated with #10 K-files. The working length was determined using an electronic apex locator and confirmed radiographically (Figure 3). Cleaning and shaping were performed with rotary NiTi files (ProTaper/ProTaper Gold; Dentsply Sirona Inc., Charlotte, NC). During instrumentation, an irregular canal wall was observed in the coronal third of the distal root, consistent with internal resorption. The resorptive defect was mechanically debrided, and a perforation was detected in the furcal area through the cervical portion of the distal root canal. Irrigation was carried out using 3% sodium hypochlorite (NaOCl), followed by 17% EDTA to remove the smear layer, and a final rinse with normal saline. Calcium hydroxide was placed as an intracanal medicament to disinfect the canals and the resorptive defect. The access cavity was temporarily sealed with Cavit, and the patient was recalled after one week.

**Figure 4:**
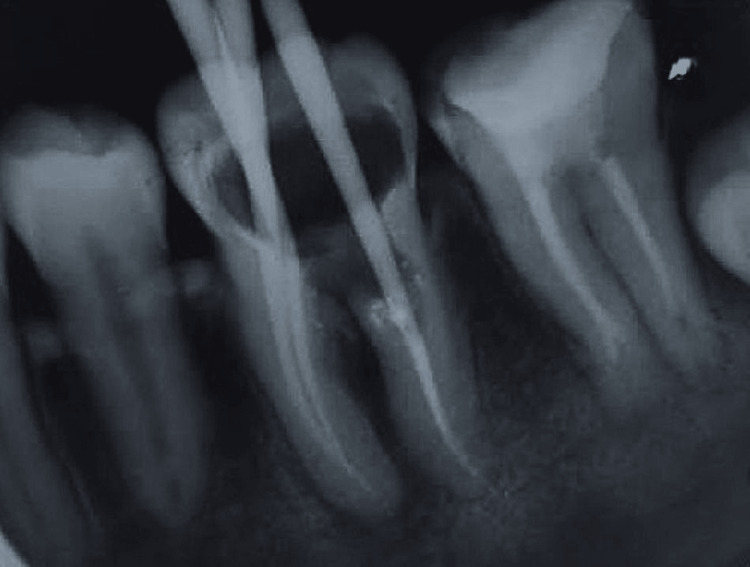
Master cone selection radiograph showing proper adaptation of gutta-percha cones to the prepared canal

At the next appointment, the temporary restoration was removed, and the canals were irrigated with 10 mL of 3% sodium hypochlorite, 10 mL of saline, and 5 mL of 17% EDTA, followed by drying with absorbent paper points. The master apical gutta-percha cone was placed, and its fit was confirmed radiographically (Figure 4). The distal canal apical to the resorptive defect, along with the mesial canals, was obturated with the corresponding gutta-percha cones and a bioceramic sealer (TotalFill, FKG, Switzerland).

A sterile collagen sheet (Kollagen-Resorb; Resorba, Nuremberg, Germany) was trimmed to the appropriate size, placed into the furcal region and resorptive defect, and gently condensed against the defect using a plugger fitted with a rubber stop. Immediately after compaction, mineral trioxide aggregate (MTA) (Angelus, Londrina, PR, Brazil) was mixed according to the manufacturer’s instructions and carefully placed into the resorptive defect and furcal region. It was then gently condensed against the collagen barrier to achieve the desired thickness, which was confirmed radiographically. Owing to the large size of the perforation and the high risk of MTA extrusion, placement of a collagen matrix prior to MTA application was deemed necessary.

**Figure 5:**
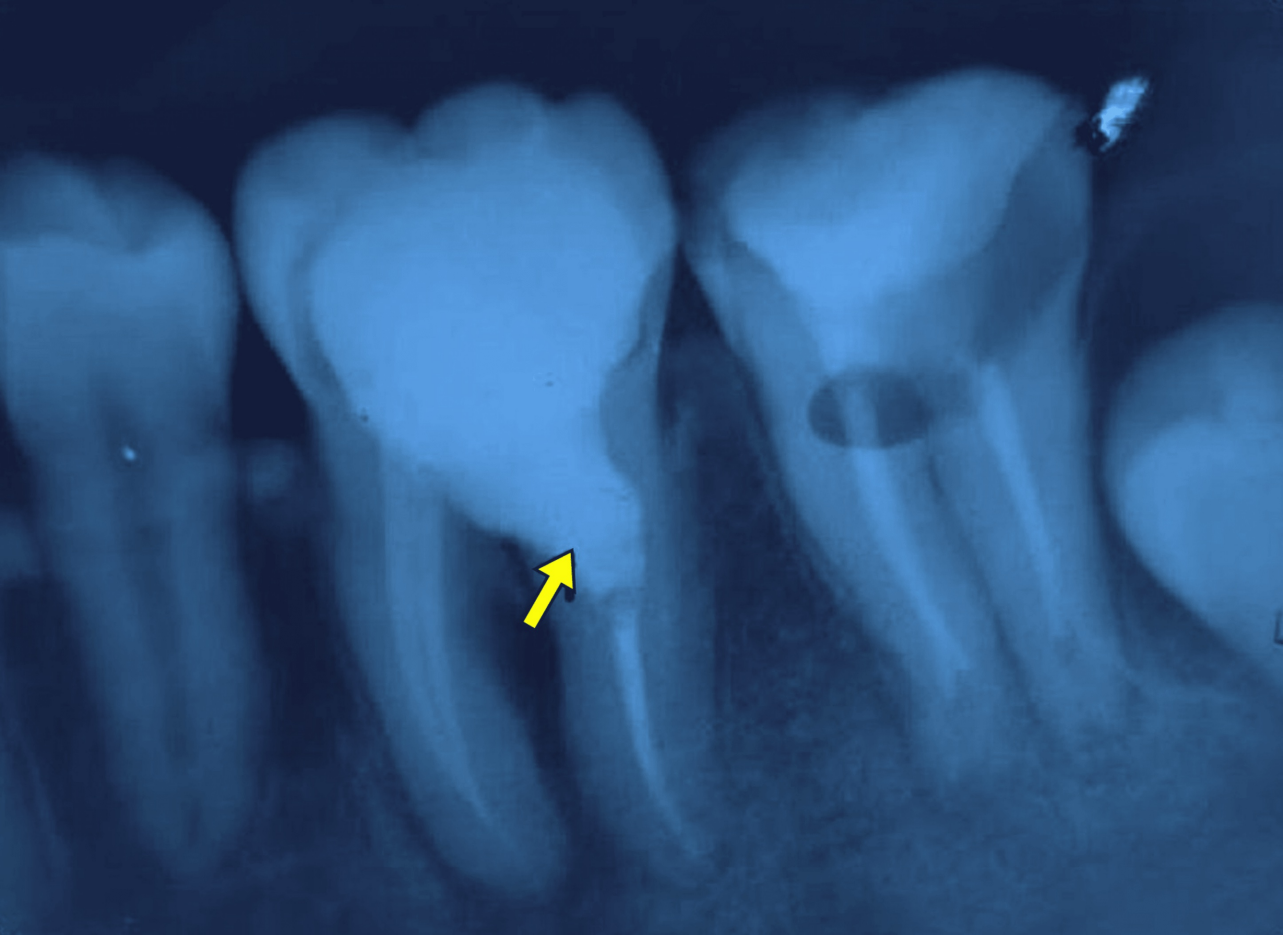
Immediate postoperative radiograph showing complete obturation of the canals and repair of the resorptive defect (arrow)

**Figure 6:**
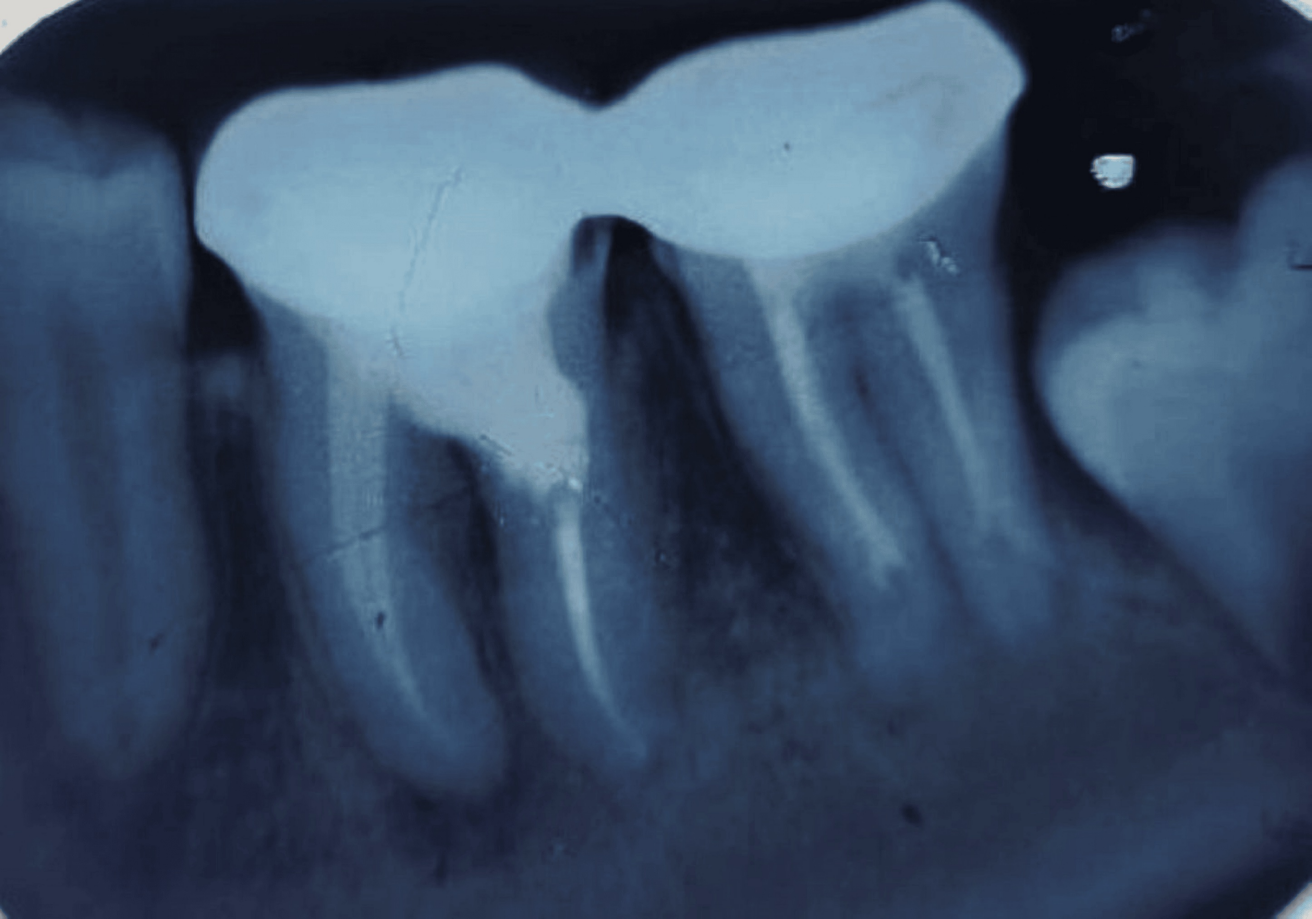
Six-month follow-up radiograph revealing resolution of periapical and furcation lesions

**Figure 7:**
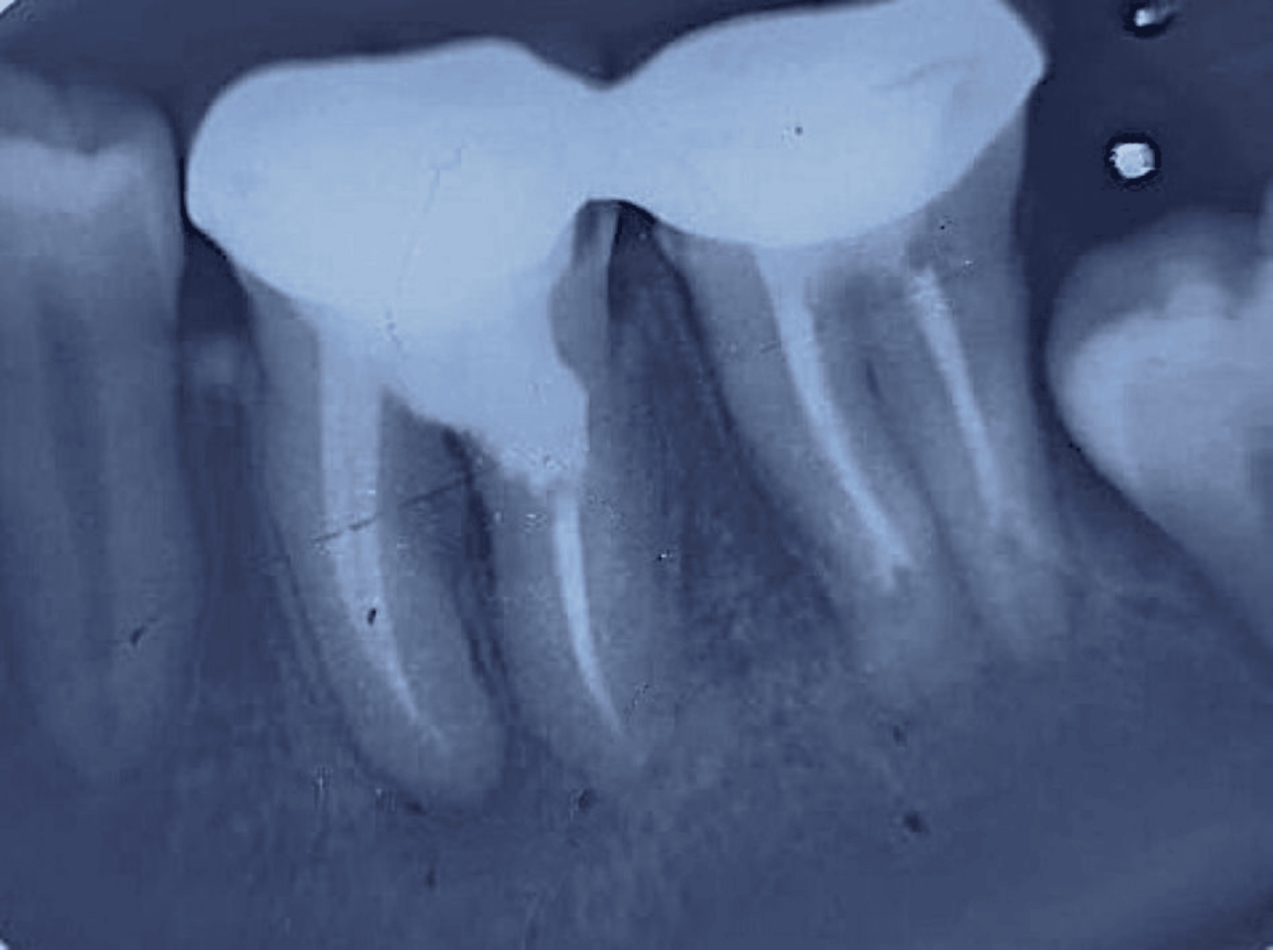
Twelve-month follow-up radiograph showing complete healing of periapical pathology and furcation lesion, and sealing of the defect is maintained

A moist cotton pellet was placed in the canal, followed by a temporary filling. The patient was recalled after 48 hours for assessment. The access cavity was restored with glass ionomer cement (Type II GC-Gold Label, GC Corporation, Tokyo, Japan). A final restoration with composite (Ivoclar Vivadent, Schaan, Liechtenstein) and a full ceramic crown bridge was then placed at the follow-up visit (Figure 5). Clinical and radiographic follow-up was conducted at six (Figure 6) and 12 (Figure 7) months postoperatively. At each follow-up, the tooth remained asymptomatic, and periodontal probing depths around the affected tooth ranged from 2-3 mm, with no increase in mobility. Pulp sensibility tests remained negative, consistent with previous endodontic treatment. Radiographic evaluation included periapical radiographs. Healing of the resorptive defect and periapical region was assessed using the periapical index (PAI) score, which improved from 4 preoperatively to 1 at 12 months, indicating complete resolution of periapical pathology.

## Discussion

Internal root resorption is a pathological process characterized by the progressive loss of dentin, and occasionally cementum, originating within the pulp space as a result of odontoclastic activity [7]. The condition is frequently asymptomatic and is often discovered incidentally during routine radiographic examinations. Reported etiological factors include trauma, chronic pulpal inflammation, restorative procedures, and idiopathic causes. Management in mandibular molars can be particularly challenging due to their complex root canal anatomy and limited accessibility, especially in cases involving perforation or furcal extension [8].

Early diagnosis and timely intervention are essential to arrest the progression of internal resorption and ensure the preservation of the natural tooth. On radiographs, internal resorption typically presents as a well-defined, symmetrical radiolucent area within the root canal system. Cone beam computed tomography (CBCT) offers superior visualization, allowing a more accurate assessment of the size, location, and extent of the lesion, particularly in complex cases involving perforations or proximity to vital anatomical structures.

In the present case, non-surgical endodontic therapy was selected as the treatment approach. The primary objectives were thorough debridement of the necrotic pulp and resorptive tissue, effective disinfection of the root canal system, and sealing of the resorptive defect to prevent reinfection. Calcium hydroxide was employed as an intracanal medicament owing to its potent antimicrobial activity and remineralizing potential [9].

Mineral trioxide aggregate (MTA) is widely recognized as an effective material for repairing resorptive defects and perforations because of its excellent biocompatibility, superior sealing ability, and ability to promote periradicular healing and cementogenesis. Its alkaline pH further enhances its antimicrobial activity, making MTA particularly valuable in managing resorptive defects associated with perforation into the periodontal tissues [10,11].

Collagen sheet (Kollagen-Resorb; Resorba, Nuremberg, Germany) is used as a matrix to provide a barrier to prevent extrusion of MTA [12].

In this case, mineral trioxide aggregate (MTA) was effectively used to fill and seal the resorptive cavity along with the associated perforation. Placement was carried out under strict isolation and magnification to ensure precision and to minimize the risk of extrusion into the periapical or furcal regions. Clinical and radiographic follow-up demonstrated satisfactory healing, with no evidence of persistent infection, pain, or mobility.

## Conclusions

Within the limitations of this single case, successful management of internal root resorption with perforation was achieved through non-surgical endodontic therapy and sealing of the defect using mineral trioxide aggregate (MTA). The favorable clinical and radiographic healing observed at the 12-month follow-up indicates that MTA can serve as a reliable material for such cases due to its excellent sealing ability and biocompatibility. However, as this report represents an isolated clinical observation, the findings should be interpreted with caution. Further case series and controlled clinical studies are recommended to substantiate these outcomes and establish stronger evidence for the long-term prognosis of similar cases.
